# Improving the Analysis of KCCQ Endpoints in Heart Failure Clinical Trials

**DOI:** 10.1007/s43441-025-00888-7

**Published:** 2025-11-10

**Authors:** Robin Myte, John Eriksson, Martin Rensfeldt, Yunyun Jiang, Ayman AL-Shurbaji, Per Nyström

**Affiliations:** 1 Biometrics, Late Stage Development, Cardiovascular, Renal and Metabolism (CVRM), BioPharmaceuticals R&D, AstraZeneca, Gothenburg, Sweden; 2 Gothenburg, Sweden

**Keywords:** Heart failure, Function and symptoms, KCCQ, Statistical analysis

## Abstract

**Background:**

The Kansas City Cardiomyopathy Questionnaire (KCCQ) is frequently used in heart failure (HF) clinical trials to evaluate treatment effects on function and symptoms. However, due to the 0–100 boundedness in KCCQ scores, conventional mean change from baseline analysis can underestimate effects for lower—and overestimate effects for higher—baselines. This study demonstrates key issues with conventional statistical methods for analyzing treatment effects on KCCQ in randomized trials and evaluates alternative statistical methods that appropriately account for 0–100 boundedness of scores.

**Methods:**

We conducted clinical trial simulations and re-analyzed a real HF randomized trial—the PRIORITIZE-HF phase II trial of sodium zirconium cyclosilicate. KCCQ change from baseline was analyzed with conventional ANCOVA models, and compared to methods that account for the 0–100 score boundedness: ANCOVA interaction models, Tobit regression, and Beta regression. Mean treatment effects and extent of bias are summarized by method.

**Results:**

There were clear baseline-dependencies in mean effects for KCCQ score change, both in simulated trials and in PRIORITIZE-HF. In the real trial, the conventional ANCOVA model mean effect on KCCQ-overall summary score was + 2.58 overall while for methods allowing effects to depend on baseline – ANCOVA interaction models and Beta regression – mean effects were over twice as large at baseline = 30 (+ 6.33 to + 6.75) and less than half at baseline = 70 (− 0.31 to + 0.89).

**Conclusions:**

Conventional analyses of treatment effects on overall mean KCCQ score changes lead to misinterpretations in clinical trials, but this can be mitigated by using methods allowing for baseline-dependent effects.

**Supplementary Information:**

The online version contains supplementary material available at 10.1007/s43441-025-00888-7.

## Introduction

Heart failure (HF) is a chronic disease associated with increased mortality and fatigue and worsened physical function, with over 60 million affected globally in 2017 [1]. While progress has been made in clinical trials for reducing hospitalizations and mortality, less focus and progress has been seen for improving patients’ function and/or symptoms [2, 3]. This is despite the obvious benefit for patients and an openness from regulatory authorities for approving drugs based on function and symptoms alone, without requiring positive results on outcomes such as hospitalizations or death [4].

For symptoms, endpoints used in clinical trials are typically based on patient-reported outcomes (PROs) in the form of self-administered questionnaires. The most commonly used is the Kansas City Cardiomyopathy Questionnaire (KCCQ), a 23-item self-administered questionnaire mapped to 7 domains which can be used to create KCCQ scores that summarize various aspects of a patient’s symptom frequency and burden [5].

However, using KCCQ for standardized evaluation of clinically meaningful change in patient health status faces important limitations [6]. A significant issue is how the baseline score limits possible changes over time given the bounded 0–100 scale. For instance, a patient starting with a baseline score of 95 can only improve by a maximum of 5 points. Thus, if the true treatment effect exceeds the difference between a patient’s baseline score and the upper limit of the scale, the observed effect in such patient groups will necessarily be underestimated. This implies that the conventional method for summarizing treatment effects on KCCQ scores in HF clinical trials—difference in mean change from baseline between treatment groups—could underestimate effects for patients with lower baseline scores and overestimate effects in patients with higher scores. These limitations pose a risk of misleading conclusions regarding treatment effects on KCCQ and their clinical meaningfulness. To our knowledge, approaches to address these issues have not been described.

In this paper, we describe the problem with using conventional statistical methods not considering the 0–100 boundedness of KCCQ scores when estimating treatment effects on these endpoints. Further, we propose more appropriate statistical methods which we demonstrate in analyses of the real PRIORITIZE-HF trial (ClinicalTrials.gov number: NCT03532009), a Phase II trial of sodium zirconium cyclosilicate (SZC) in HF [7].

## Methods

### Statistical Analysis Alternatives for KCCQ Scores

Consider a randomized controlled clinical trial with the aim to assess the treatment effect on HF function and symptoms as evaluated by the KCCQ questionnaire. The ordinal KCCQ questions range from 1–5 or 1–7, where a higher value indicates less severe symptoms or limitations. The commonly applied KCCQ endpoints, KCCQ scores such as the Overall summary score (OSS), are created by averaging sets of ordinal questions and scaling the averages to [0, 100] range. Higher scores indicate less severe symptoms and/or limitations. A general issue with all bounded scores such as KCCQ scores is that the change from baseline will be dependent on the baseline value. For example, for a patient with a baseline value of 80, the maximal possible increase is 20 and the maximal decrease is − 80.

**Fig. 1:**
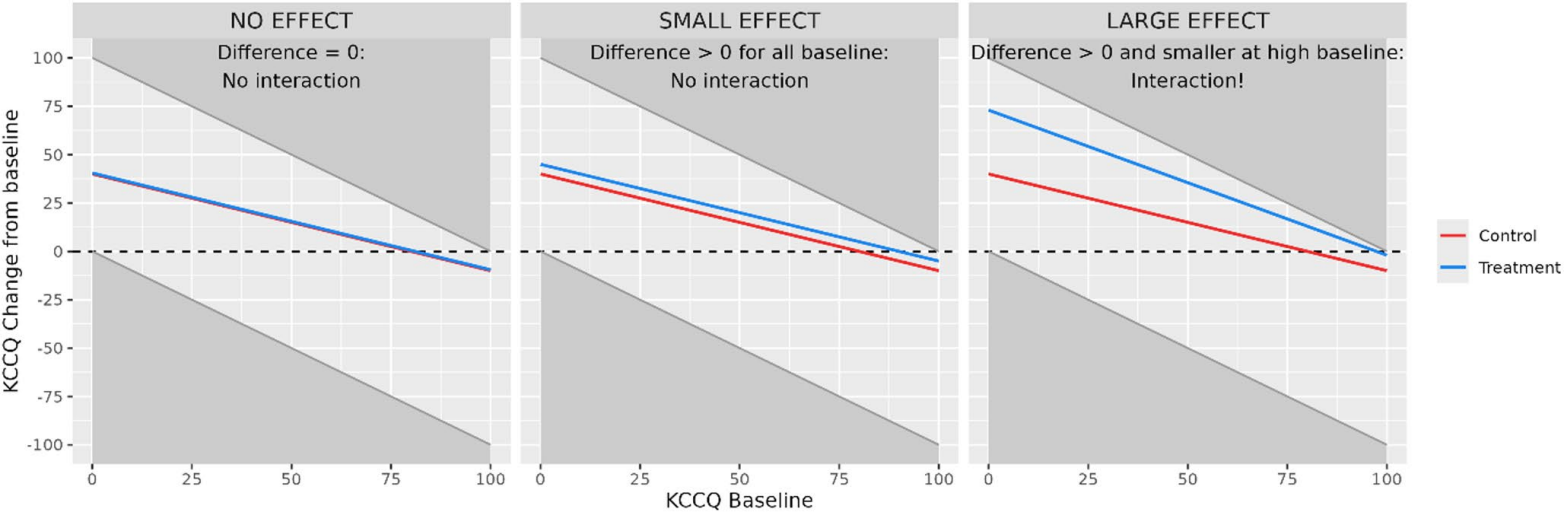
Baseline-dependency of treatment effects in conventional mean change from baseline analysis of KCCQ scores. Grey areas indicate unobservable changes caused by the boundedness of the score, lines indicate treatment group mean changes

Typically, change from baseline in KCCQ scores is analyzed using linear models such as ANCOVA or Mixed Models for Repeated Measures (MMRM) assuming normally distributed data [8–12]. These methods adjust for baseline but do not allow the effect to vary by baseline. The treatment effect is summarized as a single overall least-squares mean difference in change from baseline for all baseline values. Due to the boundedness of KCCQ scores, any large enough positive mean treatment effect will by necessity result in a baseline dependency of the effect (as illustrated in Fig. 1). For small positive treatment effects, the dependency by baseline may be too small to be detected because few patients reach the upper limit. For larger positive effects, patients at the higher end of the baseline distribution hit the 100-limit and this will occur more often in the active treatment group and the slope of the line will be different than the slope in the placebo group. An analogous argument for larger effects preventing the deterioration of symptoms applies to patients on the lower end of the range. For the same reason the assumption of constant variation across the range of covariates used in linear models (i.e., homoscedasticity) will be violated for the baseline covariate [13, 14].

As a solution to the boundedness issue, we considered three methods that—unlike the typical ANCOVA—take the issues caused by boundedness of KCCQ scores into account by allowing the estimated effect to depend on baseline:

- ANCOVA interaction model
- Tobit model
- Beta regression model

#### ANCOVA Interaction Model

The ANCOVA interaction model is the typical ANCOVA but with an added interaction term between treatment and baseline, meaning effects will be allowed to depend on baseline. The ANCOVA model for follow-up KCCQ score ${Y}_{1}$ (or equivalently change from baseline) at a single timepoint with baseline-by-treatment interaction defines the mean ${E[Y}_{1}]$ as:

$${E[Y}_{1}]={\beta }_{0}+{\beta }_{1}{Y}_{0}+{\beta }_{2}X+ {\beta }_{3}X{Y}_{0},$$

where ${Y}_{0}$ is baseline KCCQ score and $X$ a treatment indicator. This model can then be used to derive mean estimates or LS means by baseline for each treatment group, as well as measures of efficacy as differences in means by baseline. To account for heteroskedasticity, robust standard errors can be used [15]. Means within groups as well as differences between group means are preferably presented as a continuous function of baseline in a figure or tabulated at selected baseline values. A hypothesis test for overall efficacy can either be the test of mean difference equal to zero at median baseline, or a joint test of both the treatment and treatment-by-baseline interaction coefficients equal to zero against the alternative of at least one not zero. The joint test approach ensures that evidence of efficacy is detected even if the treatment effect is present only for certain baseline values or varies in magnitude across the baseline spectrum. However, when the joint test is used, it is particularly important to present estimated treatment effects by baseline values to understand the nature of potential baseline-by-treatment interaction.

#### Tobit Model

The Tobit model, sometimes also referred to as censored regression, is a linear model like ANCOVA but relies on the idea that the observed range of the dependent variable of interest is censored [16]. Applied to KCCQ scores, this means that the latent symptom(s) variable we aim to improve is unbounded and normally distributed, but the observed KCCQ score is censored at 0 and 100. This implies e.g., that a patient at 100 is not necessarily at the same “symptom level” as another patient with 100 (or within the same patient with 100 at different timepoints). A linear Tobit model for KCCQ score values is defined as:

$${E[Y}_{1}^{*}]={\beta }_{0}+{\beta }_{1}{Y}_{0}+{\beta }_{2}X+{\beta }_{3}X{Y}_{0},$$

where ${Y}_{1}^{*}$ is the latent variable, observed as ${Y}_{1}$:

$$ Y_{1} = \begin{array}{*{20}c} {0\,if\,Y_{1}^{*} } & { \le \,0,} & {} \\ {Y_{1}^{*} \,if\,0\, < \,Y_{1}^{*} } & { < \,100,} & {} \\ {100\,if\,Y_{1}^{*} } & { \ge \,100.} & {} \\ \end{array} $$

The baseline by treatment interaction term is included to allow for a baseline-dependent effect. Although an interaction should not be present due to the boundedness of the scale as it is handled by modeling the latent unbounded variable, the effect of the drug may still differ for patients at lower vs higher baselines and thus it is reasonable to still include an interaction term. The Tobit model estimates can be used to provide efficacy estimates and hypothesis tests in a similar manner as for the ANCOVA interaction model.

#### Beta Regression

**Fig. 2:**
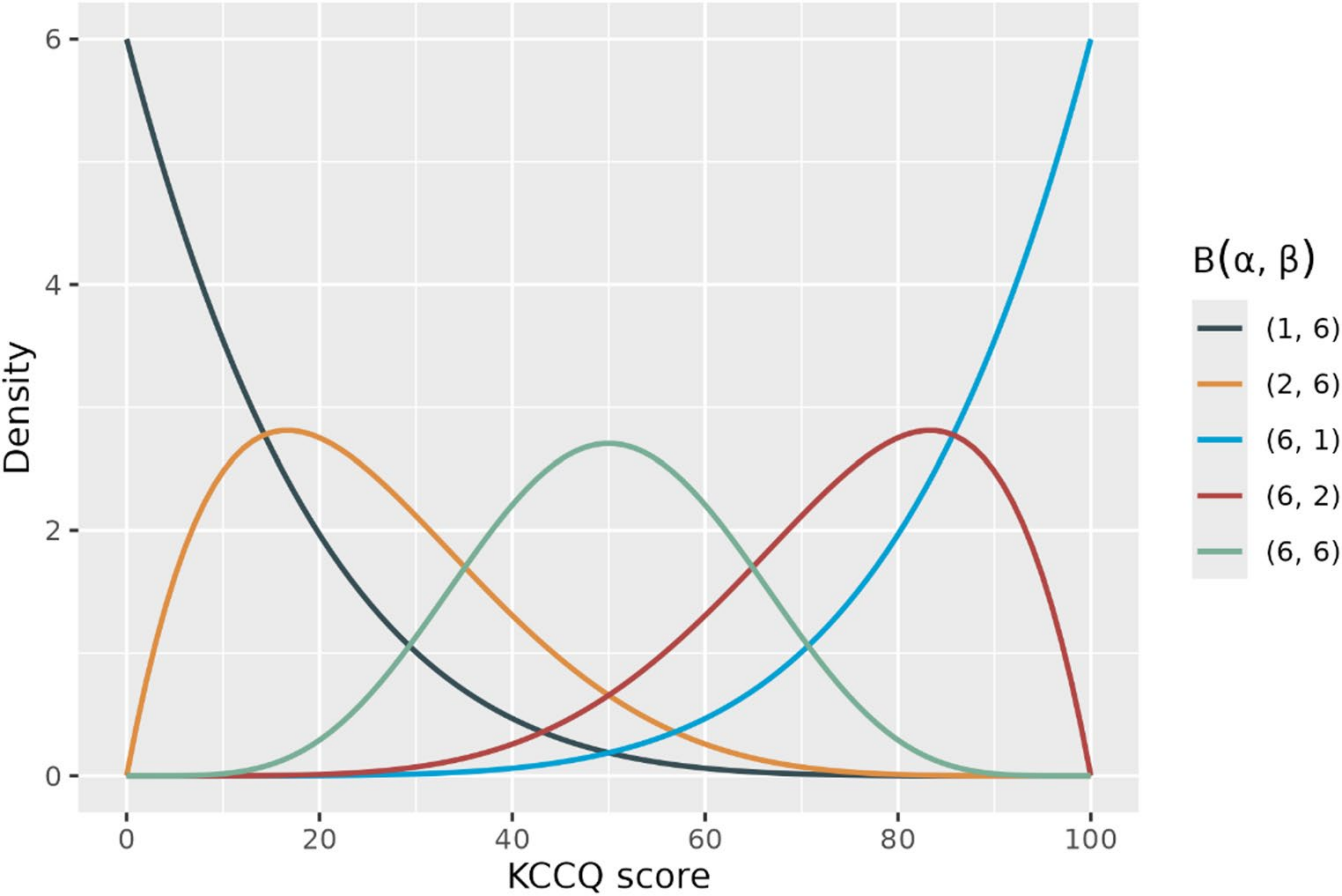
Examples of possible distributions for beta distributed KCCQ score as characterized by $\alpha$ and $\beta$ parameters (multiplied by 100 from original beta variable)

Unlike the ANCOVA interaction and the Tobit models that assume normal distribution of the dependent variable, beta regression handles 0–100 boundedness directly and explicitly by assuming that the data is continuous and beta distributed between 0 and 1 (equivalent to the 0–100 KCCQ bounds scaled by 1/100) [17]. KCCQ scores are not actually continuous on the 0–100 interval. However, as most common scores are based on a larger number of questions, continuous scores should be a reasonable approximation. The beta distribution is characterized by two parameters, $\alpha$ and $\beta$, often referred to as shape and scale and can assume several different distributional shapes as shown in the Fig. 2. This makes it a very flexible distribution for analysis. In beta regression the distribution is reparametrized and described by two parameters, a mean parameter which equals the expected value: $\mu =E\left[{Y}_{1}\right]=\frac{\alpha }{\alpha +\beta }$, and a precision parameter $\sigma =\alpha +\beta .$ We define the model for the $\mu$ parameter:

$$g(\mu )={\beta }_{0}+{\beta }_{1}{Y}_{0}+{\beta }_{2}X+ {\beta }_{3}X{Y}_{0},$$

where $g(.)$ is a link function, typically the log or logit (i.e., $\mathrm{log}(\frac{\mu }{1-\mu })$) function. The variance of ${Y}_{1}$ is dependent on both the mean and sigma parameters, $V\left[{Y}_{1}\right]=\mu \left(1-\mu \right)/(1+\sigma )$, and therefore directly incorporates heteroskedasticity. An extended version of the model also defines a similar model for the $\sigma$ parameter. Similar to the Tobit model, an interaction due to boundedness will not be present, but a baseline-by-treatment interaction term is still included to capture possible differences in drug effect across baseline values.

An issue with the beta regression models is that the beta distribution only has support on the open interval (0,1), while KCCQ can also take the values 0 and 100. A possible data transformation is to map zeros to $\delta$ and 100 s to $100(1-\delta )$, for some small $\delta$. Another solution is to apply the 0/1 inflated beta regression which additionally models $P(Y=0)$ and $P(Y=1)$ similarly as $\mu$ or with a simplified model (typically with a log link). The best choice of model, i.e., whether the standard, extended (with $\sigma$ model), or 0/1 inflated model should be used, depends on the data (e.g., expected scores at bounds 0 and 100). When the model needs to be pre-specified, a recommended approach is to assume the more complex model i.e., the 0/1 inflated model and potentially simplify based on model fit in study as data accrue or information from other studies.

Estimates of $\mu$ within each treatment group can be obtained by applying the inverse link function (${g}^{-1}(\mu )$, e.g., the inverse logit) to the model’s linear predictor using the estimated model coefficients, converting fitted means back to the original outcome scale. Mean change from baseline can then be calculated by subtracting the relevant baseline value from the back-transformed means, and differences between treatment groups across baseline values can be obtained by taking the difference in these means. Corresponding confidence intervals can be derived using e.g., non-parametric bootstrap, while hypothesis testing can be based on model coefficient tests.

### Simulation

 To show the impact of KCCQ score boundedness on traditional mean change analysis, we simulate a large representative 1:1 randomized clinical trial on KCCQ assuming scores are naturally bounded through the beta distribution. KCCQ data is simulated for N = 4000 patients (2000 per arm) from a 0–1 beta distribution, which is then multiplied by 100 to get 0–100 scores. The average treatment effect is assumed to be large, with an overall > 10 increase in mean score in treatment vs control, and the baseline distribution is one of two scenarios: low (mean = 40) or high (mean = 60), and variance was $\sigma =0.4$. Further details of the simulations are provided in the Supplementary methods.

### Application in real clinical trial

 To demonstrate the proposed statistical methods, we conducted an analysis of KCCQ data in the PRIORITIZE-HF trial. PRIORITIZE-HF was an international double-blind randomized clinical trial to evaluate the benefits and risks of using the potassium-lowering agent SZC to intensify RAAS inhibitor treatment without inducing clinically significant hyperkalemia in patients with symptomatic HF with reduced ejection fraction [7]. Patients were randomly assigned to receive SZC 5 g or placebo once daily for 12 weeks in a double-blind fashion, and KCCQ was an exploratory endpoint assessed at baseline and at 12 weeks. Baseline and follow-up study characteristics are presented in Supplementary Table 1, and further details on the analyses are provided in the Supplementary methods. Data underlying the findings described in this manuscript can be obtained in accordance with AstraZeneca’s data sharing policy described at https://astrazenecagrouptrials.pharmacm.com/ST/Submission/Disclosure.

## Results

### Simulations

**Fig. 3:**
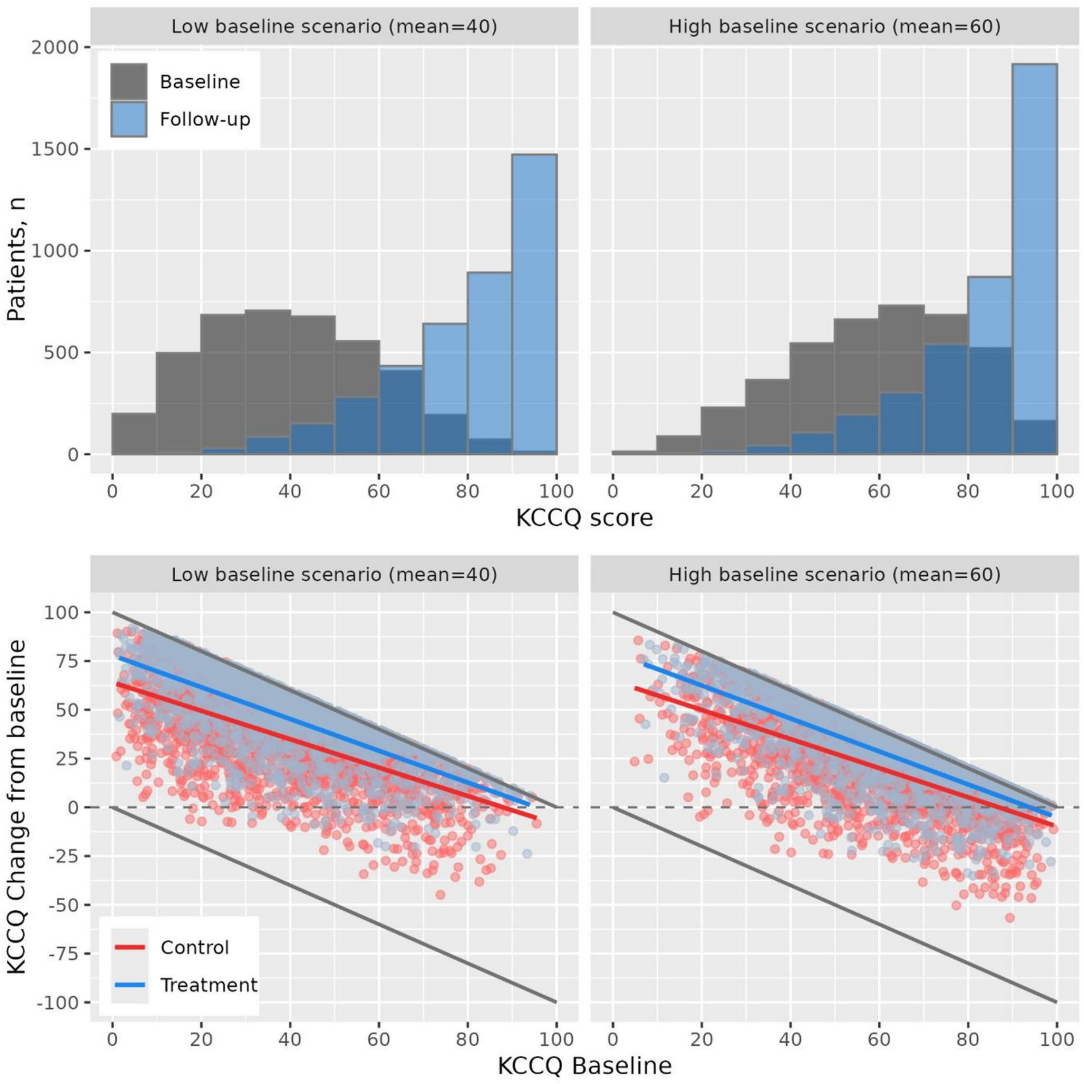
KCCQ distributions and change from baseline in simulated randomized trials. Distributions for two N = 4000 simulated trials with a large effect on KCCQ and either a low or high baseline KCCQ distribution, respectively. Grey lines indicate bounds of the scores, colored lines represent mean regression lines per treatment group

 KCCQ distribution and mean change from baseline from two representative simulated trials are presented in Fig. 3. For an unbounded normally distributed endpoint, a positive average effect shifts the distribution upwards without affecting the shape of the distribution. For a bounded endpoint like KCCQ scores, this is not the case since a positive shift leads to more patients reaching the 100-ceiling. This causes a change in both the location and shape of the distribution, which is seen clearly in the simulated trial histograms of baseline and follow-up scores. The pattern of baseline dependency on change from baseline at follow-up is also clear, with smaller changes at higher baseline values and vice versa. As there is a true treatment effect, patients reaching the 100-ceiling occurs more often in the treatment group meaning an increasingly narrowing difference between treatment group means going from low to high baseline.

Analyzing treatment effects in these simulated trials with traditional overall means gives treatment effects of KCCQ change of approximately 10. But if one instead allows effects to depend on baseline through baseline-by-treatment effect interaction—this gives mean effects of 6–14 from baseline 10–90 with significant tests for interaction (interaction *p*-value < 0.001). Although the two trials are simulated assuming the same true effect, estimated effects at higher baselines are somewhat larger in the low baseline trial (5.2) than the high baseline trial (4.6)—which comes from fewer patients reaching the 100-ceiling in the low baseline scenario. The opposite is true at lower baselines, as more patients in the low baseline group hit the 0-floor.

Tobit models and Beta regression naturally incorporate boundedness in the modeling, and since our simulation model does not assume a treatment-by-baseline interaction for other reasons, the resulting Tobit and Beta estimates in our simulated trials remain essentially unchanged regardless if a treatment-by-baseline interaction term is included in these models or not (Supplementary Fig. 1). The ANCOVA model, on the other hand, requires the interaction term to allow effects to depend on baseline.

For ANCOVA interaction models, standard errors were smallest near the mean baseline value and become larger at the extremes of the baseline distribution which naturally reflects the increased uncertainty where data is sparse (Supplementary Fig. 2). In the high baseline scenario, however, standard errors at the upper end are smaller compared to the low baseline scenario, as more patients reach the upper 100-ceiling. Although only small differences were observed between unadjusted and robust standard error estimates, robust SEs tended to be slightly lower at higher baseline values and slightly higher at lower baseline values. This pattern is consistent with the characteristics of a bounded outcome, where variability decreases near the outcome ceiling.

### Application in PRIORITIZE-HF

**Fig. 4:**
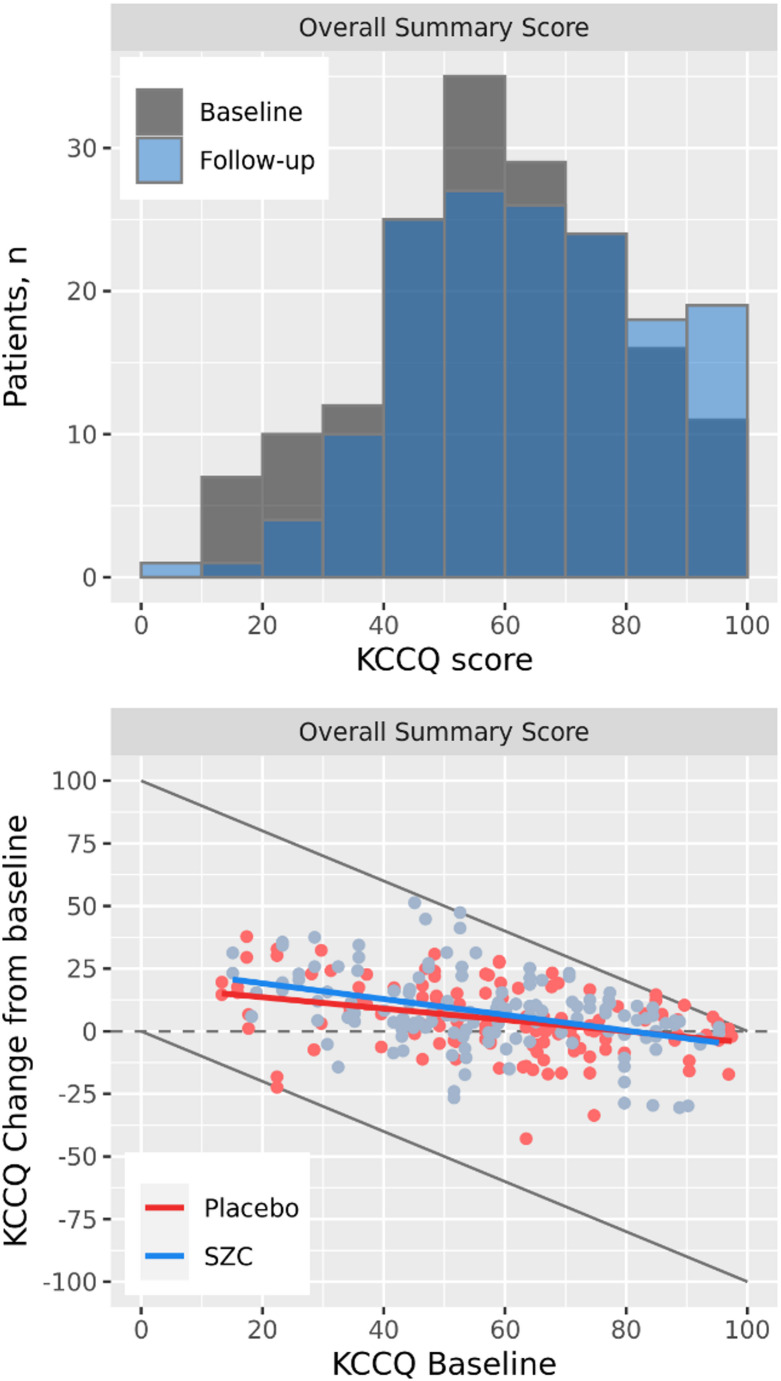
KCCQ Overall Summary Score distributions and individual changes from baseline in PRIORITIZE-HF. Grey lines indicate bounds of the scores, colored lines represent mean regression lines per treatment group

Distribution and individual KCCQ Overall Summary scores in PRIORITIZE-HF are shown in Fig. 4. Although relatively few patients hit the lower and upper bounds of KCCQ in this study, the same pattern of baseline dependency on change from baseline as in the simulated trials is observed.

**Fig. 5:**
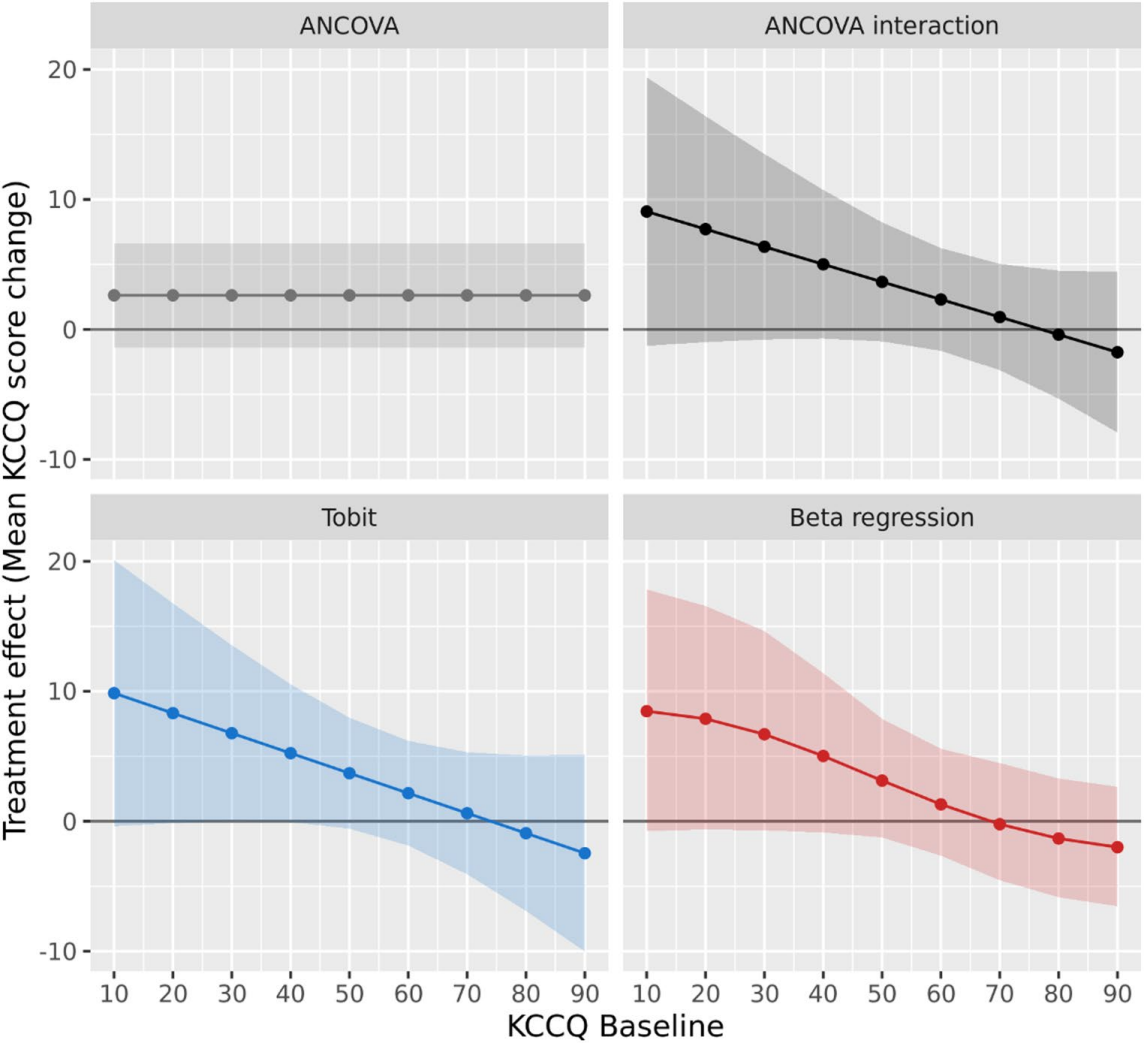
Mean treatment effects for KCCQ Overall Sumary Score change from baseline in PRIORITIZE-HF. Treatment effects represent differences in mean change from baseline at 12 weeks between treatment groups. Shaded areas represent 95% confidence intervals. Mean effects > 0 favors SZC treatment

The estimated effect for KCCQ Overall Summary score are vizualised as a function of baseline for all methods in Fig. 5. The mean effects from the conventional ANCOVA are constant across baseline since it does not allow the effect to depend on baseline. At the mean baseline = 58, treatment effects were similar across methods: 2.62 for typical ANCOVA and 2.57, 2.47, and 1.65 for ANCOVA interaction, Tobit, and Beta regression respectively (Table 1). The underestimation of effects at lower baseline scores and overestimation at higher scores for the conventional ANCOVA is clear when comparing to results from methods allowing the effect to depend on baseline. Lower estimates of the treatment effect were seen for KCCQ change at baseline = 70 (− 0.31 to 0.89 across estimators) and larger estimates at baseline = 30 (6.33 to 6.75 across estimators). Similar patterns were seen also for other scores; KCCQ Total Symptom Score and KCCQ Clinical Summary Score (Supplementary Fig. 3). Given the few patients with observed follow-up scores at the bounds 0 or 100, results between methods were similar. For instance, Tobit estimates in the SZC arm are the same as ANCOVA estimates since there are no patients with observed score at the bounds in that group. The overall pattern of baseline-dependent effect can be seen for all methods, with the difference that beta regression also incorporates nonlinear patterns in the effect by baseline. The impact of allowing for nonlinear influence of baseline is also evident in within-arm change from baseline estimates in Table 1, where estimates are somewhat higher for beta regression compared to the linear ANCOVA and Tobit models—also at the mean. This likely reflects the limitation of linear models, which constrains the relationship between change and baseline to be strictly increasing or decreasing, whereas beta regression is able to capture more complex, nonlinear associations.

**Table 1:** KCCQ Overall summary score change from baseline results in PRIORITIZE-HF

	Estimate	Comparison of treatment groups					
Estimator	Baseline	Placebo (N = 86, n = 78)	SZC (N = 83, n = 76)	Estimate	95% CI	*p*-value	Interaction* p*-value
ANCOVA	30	11.92	14.55	2.62	–1.37, 6.62	0.200	–
	58 (mean)	5.33	7.95	2.62	−1.37, 6.62		
	70	2.50	5.13	2.62	−1.37, 6.62		
ANCOVA interaction	30	10.06	16.42	6.36	−0.78, 13.50	0.091	0.141
	58 (mean)	5.20	7.77	2.57	−1.45, 6.59		
	70	3.11	4.06	0.95	−3.14, 5.04		
Tobit	30	9.64	16.42	6.78	0.01, 13.55	0.065	0.128
	58 (mean)	5.30	7.77	2.47	−1.53, 6.47		
	70	3.44	4.06	0.62	−4.07, 5.31		
Beta regression	30	10.35	17.05	6.69	−0.97, 14.03	0.098	0.171
	58 (mean)	7.18	8.83	1.65	−2.79, 6.17		
	70	4.33	4.11	-0.23	-4.77, 4.16		

Estimates represent means for change from baseline and differences in the same between treatment groups for the comparison derived from the fitted models. Deaths were handled using a while-alive approach, i.e., for patients who die the last measurement prior to death was used in the analysis (n = 1). N Number of subjects per treatment group; n Number of subjects in analysis; SCZ, sodium zirconium cyclosilicate

## Discussion

In this paper, we provide appropriate statistical methodology for HF clinical trials assessing function and symptoms using KCCQ-based endpoints. We argue and demonstrate in simulations and a real clinical trial the need to consider the 0–100 boundedness of KCCQ scores and present effects by baseline score by including baseline-by-treatment interaction in linear models or by using methods not assuming a normal distribution like beta regression.

As demonstrated in both our simulated trials and the PRIORITIZE-HF trial, failing to appropriately handle the boundedness of KCCQ scores often leads to misinterpretations of the data since any large enough mean treatment effect will always be dependent on the baseline score. This happens because patients with higher baseline scores have less room for improvement before reaching the upper 100-ceiling, so the observed treatment effect is constrained by how close their baseline is to the maximum score. Consequently, summarizing the treatment effect as a single difference in mean change from baseline cannot convey the expected treatment effect on the individual patient level. It is therefore, arguably, of limited value to patients and clinicians. Interestingly, in two recent HF trials—the FINEART-HF and STEP-HFpEF trials [11, 12]—while the main KCCQ analyses were conducted traditionally using overall means, supplementary KCCQ analyses by baseline have also been published separately [13, 14], possibly indicating a growing awareness of the issue.

Using ANCOVA with baseline-by-treatment interaction solves the boundedness-issue by allowing the effect to depend on baseline, but it remains unclear whether robust standard errors can fully adjust for the baseline dependency in variance (i.e., heteroskedasticity) that also comes from the bounded scale. Unadjusted standard errors are expected to overestimate uncertainty near the outcome ceiling, and while robust standard errors appear to mitigate this effect slightly in our simulations, it is still uncertain whether they can fully resolve the issue; further research is needed. Nonlinear baseline-dependency of the treatment effect can be explicitly modeled in ANCOVA models, but this can be difficult to pre-specify in a blinded trial. It is possible to use a more data-driven approach to estimate nonlinear effects, for instance using restricted cubic splines as was done in FINEART-HF [14]. However, such a model still assumes linearity in the ends of the baseline distribution to avoid overfitting, which is closest to the bounds where the nonlinearity of KCCQ baseline-by-treatment is likely to be most pronounced, meaning the risk remains that estimates and variances are biased. Another solution is using the Tobit model assuming the KCCQ score is a censored observation of an unbounded latent normally distributed variable, but the interpretation using this method is not straightforward. Beta regression represents the most flexible option to solve the boundedness issue by assuming a more plausible true distribution for the scores than the normal distribution—the naturally bounded beta distribution. This method allows for both nonlinear effects by baseline and heteroskedasticity. The price of this flexibility is added complexity in the model. Beta regression requires the specification of models for up to four population parameters which may be difficult to pre-specify (the mean, variance, and probabilities of hitting 0-floor and 100-ceiling), instead of just modeling one as in the ANCOVA and Tobit model (the mean). Furthermore, assumptions for statistical power calculations are not straightforward and warrant further study of previous HF studies with varying degrees of treatment effect on KCCQ scores. In summary, we consider both the ANCOVA interaction model and Beta regression as appropriate methods for analysing KCCQ scores, with beta regression being the most flexible but also the more complex option.

Note that the purpose of this paper is to discuss alternative approaches to analyse KCCQ, not to evaluate the efficacy of SZC. Due to differences in included patients for consent reasons, timepoint assessed, and analysis methods used the estimated treatment effect of SZC on KCCQ endpoints in this paper differs slightly from the previously published results [7]. This should not be seen as contradictory. It also is important to note that while the inherent baseline dependency of effects from the bounded KCCQ scores means numerically smaller effects at higher baselines, this does not mean such changes are not clinically meaningful for a patient. Clinical meaningfulness thresholds for mean effects on KCCQ can, for instance, be based on data using anchor-based methods [18]. For the same reason treatment effects on KCCQ must depend on baseline due to the bounded scale, clinical meaningfulness thresholds must also depend on baseline and should be considered in their derivations.

Some limitations of this study should be acknowledged. First, our findings rely on simulations and a single clinical trial (PRIORITIZE-HF). While KCCQ score treatment effects are inherently baseline-dependent due to the scale’s 0–100 range, larger studies with varied baselines are needed for confirmation. Second, the use of beta regression in clinical trials requires further research to assess its feasibility and implications for statistical power, especially with varying treatment effects on KCCQ scores. Nonetheless, the simpler ANCOVA model with baseline-by-treatment interaction is likely sufficient for most HF trials, offering reduced bias vs currently used methods, while maintaining ease of use. Finally, this study did not address other methods such as hierarchical endpoint methods that combine clinical events and PROs [19]. While the effect on such endpoints can be relevant, it responds to a different scientific question, namely that of the effect on the composite outcome of the function and/or symptoms and death or events combined. A particular problem in interpreting such objectives arises when the groups are unbalanced in opposite directions on clinical event incidences and the selected PROendpoint [20]. As such, we did not consider these methods in this paper, where focus was trial objectives specifically targeting effects on improvement in function and symptoms alone.

## Conclusion

In conclusion, conventional analyses of mean changes in KCCQ scores in clinical trials can lead to misinterpretations of the treatment effect due to the 0–100 boundedness. Primary analysis of KCCQ scores in clinical trials should therefore consider using methods allowing mean KCCQ score effects to depend on baseline, such as baseline-interaction models or beta regression. Our findings may also have implications for other disease areas using similar PRO-based endpoints.

## Supplementary Information

Below is the link to the electronic supplementary material.

Supplementary Material 1

## Data Availability

Data underlying the findings described in this manuscript can be obtained in accordance with AstraZeneca’s data sharing policy described at https://astrazenecagrouptrials.pharmacm.com/ST/Submission/Disclosure.

## References

[1] Groenewegen A, Rutten FH, Mosterd A, Hoes AW. Epidemiology of heart failure. Eur J Heart Fail. 2020;22:1342–56. 32483830 10.1002/ejhf.1858 PMC7540043

[2] Butler J, Hamo CE, Udelson JE, Pitt B, Yancy C, Shah SJ, et al. Exploring new endpoints for patients with heart failure with preserved ejection fraction. Circ Heart Fail. 2016. 10.1161/CIRCHEARTFAILURE.116.003358. 27756791 10.1161/CIRCHEARTFAILURE.116.003358

[3] Fiuzat M, Lowy N, Stockbridge N, Sbolli M, Latta F, Lindenfeld J, et al. Endpoints in heart failure drug development: history and future. JACC Heart Fail. 2020;8:429–40. 32278679 10.1016/j.jchf.2019.12.011

[4] FDA. Discussion document for patient-focused drug development public workshop on guidance 4: Incorporating clinical outcome assessments into endpoints for regulatory decision-making. In: Patient-focused drug development. U.S. Food and drug administration; 2019.

[5] Spertus JA, Jones PG, Sandhu AT, Arnold SV. Interpreting the Kansas City cardiomyopathy questionnaire in clinical trials and clinical care: JACC state-of-the-art review. J Am Coll Cardiol. 2020;76:2379–90. 33183512 10.1016/j.jacc.2020.09.542

[6] Stogios N, Fezza G, Wong JV, Ross HJ, Farkouh ME, Nolan RP. Current challenges for using the Kansas City cardiomyopathy questionnaire to obtain a standardized patient-reported health status outcome. Eur J Heart Fail. 2021;23:205–7. 33619798 10.1002/ejhf.2139 PMC8049137

[7] Tardif JC, Rouleau J, Chertow GM, Al-Shurbaji A, Lisovskaja V, Gustavson S, et al. Potassium reduction with sodium zirconium cyclosilicate in patients with heart failure. ESC Heart Fail. 2023;10:1066–76. 36564955 10.1002/ehf2.14268 PMC10053160

[8] Butler J, Filippatos G, Jamal Siddiqi T, Brueckmann M, Bohm M, Chopra VK, et al. Empagliflozin, health status, and quality of life in patients with heart failure and preserved ejection fraction: the EMPEROR-preserved trial. Circulation. 2022;145:184–93. 34779658 10.1161/CIRCULATIONAHA.121.057812 PMC8763045

[9] Kosiborod MN, Jhund PS, Docherty KF, Diez M, Petrie MC, Verma S, et al. Effects of dapagliflozin on symptoms, function, and quality of life in patients with heart failure and reduced ejection fraction: results from the DAPA-HF trial. Circulation. 2020;141:90–9. 31736335 10.1161/CIRCULATIONAHA.119.044138 PMC6964869

[10] Lewis EF, Claggett BL, McMurray JJV, Packer M, Lefkowitz MP, Rouleau JL, Liu J, Shi VC, Zile MR, Desai AS, et al. Health-related quality of life outcomes in PARADIGM-HF. Circ Heart Fail. 2017;10. 10.1161/CIRCHEARTFAILURE.116.003430 28784687

[11] Kosiborod MN, Abildstrøm SZ, Borlaug BA, Butler J, Rasmussen S, Davies M, et al. Semaglutide in patients with heart failure with preserved ejection fraction and obesity. N Engl J Med. 2023;389:1069–84. 37622681 10.1056/NEJMoa2306963

[12] Solomon SD, McMurray JJV, Vaduganathan M, Claggett B, Jhund PS, Desai AS, et al. Finerenone in heart failure with mildly reduced or preserved ejection fraction. N Engl J Med. 2024;391(16):1475–85. 39225278 10.1056/NEJMoa2407107

[13] Kosiborod MN, Verma S, Borlaug BA, Butler J, Davies MJ, Jon Jensen T, et al. Effects of semaglutide on symptoms, function, and quality of life in patients with heart failure with preserved ejection fraction and obesity: a prespecified analysis of the STEP-HFpEF trial. Circulation. 2024;149:204–16. 37952180 10.1161/CIRCULATIONAHA.123.067505 PMC10782938

[14] Yang M, Henderson AD, Talebi A, Atherton JJ, Chiang C-E, Chopra V, et al. Effect of finerenone on the KCCQ in patients with HFmrEF/HFpEF: a prespecified analysis of FINEARTS-HF. J Am Coll Cardiol. 2025;85(2):120–213. 39520455 10.1016/j.jacc.2024.09.023

[15] MacKinnon JG, White H. Some heteroskedasticity-consistent covariance matrix estimators with improved finite sample properties. J Econom. 1985;29:305–25.

[16] Tobin J. Estimation of relationships for limited dependent variables. Econometrica. 1958;31:24–36.

[17] Kieschnick R, McCullough BD. Regression analysis of variates observed on (0, 1): percentages, proportions and fractions. Stat Model. 2003;3:193–213.

[18] Butler J, Khan MS, Mori C, Filippatos GS, Ponikowski P, Comin-Colet J, et al. Minimal clinically important difference in quality of life scores for patients with heart failure and reduced ejection fraction. Eur J Heart Fail. 2020;22:999–1005. 32239794 10.1002/ejhf.1810

[19] Ferreira JP, Jhund PS, Duarte K, Claggett BL, Solomon SD, Pocock S, et al. Use of the win ratio in cardiovascular trials. JACC Heart Fail. 2020;8:441–50. 32466836 10.1016/j.jchf.2020.02.010

[20] Permutt T, Li F. Trimmed means for symptom trials with dropouts. Pharm Stat. 2017;16:20–8. 27523396 10.1002/pst.1768

