# Supplementary material for: Improving the Analysis of KCCQ Endpoints in Heart Failure Clinical Trials
Source: Ther Innov Regul Sci. 2025 Nov 10;60(2):326–35. doi: 10.1007/s43441-025-00888-7 (PMC12946294; doi:10.1007/s43441-025-00888-7)
Supplement: Supplementary file 1 — Supplementary Material 1 [file 43441_2025_888_MOESM1_ESM.docx]

S1. Supporting information

*Improving the Analysis of KCCQ Endpoints in Heart Failure Clinical Trials*
Myte et al

Contents

[**Supplementary methods** 2](#_Toc210637621)

[Simulations 2](#_Toc210637622)

[PRIORITIZE-HF analyses 2](#_Toc210637623)

[References 3](#_Toc210637624)

[**Supplementary Tables and Figures** 4](#_Toc210637625)

[**Supplementary Table 1** 4](#_Toc210637626)

[**Supplementary Figure 1** 5](#_Toc210637627)

[**Supplementary Figure 2** 6](#_Toc210637628)

[**Supplementary Figure 3** 7](#_Toc210637629)

## **Supplementary methods**

### Simulations

We simulated 1:1 randomized KCCQ clinical trials assuming KCCQ scores are naturally bounded through the beta distribution. KCCQ data was simulated from a 0-1 beta distribution, then multiplied by 100 and rounded to get 0-100 scores. Baseline values were simulated directly assuming a given scenario mean (low=40 or high=60), and follow-up values were simulated assuming the mean is determined by a linear predictor linked through the logit function – i.e., a Beta regression model similar to what is described in section above. Specifically, $g(\mu)=\beta_{0}+\beta_{1}y_{0}+\beta_{2}X$, where $\beta_{0}=0.5$, $\beta_{1}=1.5$*,* $\beta_{2}$=0.7 which approximately corresponds to a true treatment effect on change from baseline of 10 at baseline=50. The variance was set to $\sigma=0.4$ for both baseline and follow-up, based on estimates in PRIORITIZE-HF. Each simulated trial consists of N=4000 patients (2000 per arm).

### PRIORITIZE-HF analyses

In PRIORITIZE-HF, KCCQ was an exploratory endpoint with planned evaluation at baseline, at 12 weeks, and 16 weeks.^1^ Due to challenges in participant management related to the COVID-19 pandemic, the study was prematurely terminated with 182 randomized patients of which n=169 where available for this analysis. Some patients attended planned visits earlier than anticipated, this paper will focus on KCCQ data from the baseline and week 12 follow-up visit regardless of when the follow-up visit occurred in time. Study baseline and follow-up characteristics are presented in **Supplementary Table 1.**

Patients who died prior to the follow-up visit were handled with the while-alive strategy in the analyses – i.e., using the latest value observed prior to death as the follow-up value (n=1 patient). Missing baseline KCCQ data were imputed with the mean for scores and mode for individual questions (n=2 patients) and patients with missing KCCQ data at follow-up for non-death reasons were excluded prior to analysis (n=14 patients), leaving n=155 patients for the analyses.

All analyses were made in R 4.1.0.^2^

For KCCQ score analyses, all models include KCCQ score result at 12 weeks as the dependent variable and baseline, treatment, and baseline-by-treatment as covariates unless otherwise specified. ANCOVA interaction models with robust standard errors were fitted using the lm_robust function in the estimatr package, using the heteroskedasticity-consistent 2 (HC2) standard error estimator. For comparison, ANCOVA models without interaction term and robust standard errors were also fitted using the lm function. Tobit models were fitted using the censReg function in the censReg package,^3^ with the same covariates as the ANCOVA model. Beta regression was fitted using the gamlss function in the gamlss package.^4^ A 0/1-inflated model was used, where the $\mu$ parameter was modelled using a logit link. Remaining parameters σ, P(Y=1), and P(Y=0) were modelled by baseline and treatment.

Efficacy estimates using each of the described methods are presented as means for change from baseline, derived using estimated coefficients and covariance matrices. Beta-regression estimates were back-transformed to the original scale by taking the inverse logit. CIs for beta-regression estimates were based on non-parametric bootstrap (R=500). Presented p-values represent coefficient tests the model ($\mu$ coefficients for beta regression).

### References

1. Tardif JC, Rouleau J, Chertow GM, Al-Shurbaji A, Lisovskaja V, Gustavson S, Zhao Y, Bouabdallaoui N, Desai AS, Chernyavskiy A, et al. Potassium reduction with sodium zirconium cyclosilicate in patients with heart failure. *ESC Heart Fail*. 2023;10:1066-1076.

2. RCoreTeam. *R: A language and environment for statistical computing. R Foundation for Statistical Computing, Vienna, Austria.* [*https://www.R-project.org/*](https://www.R-project.org/)*.* 2018.

3. Henningsen A. Estimating Censored Regression Models in R using the censReg Package. Paper/Poster presented 2012;

4. Stasinopoulos DM, Rigby RA. Generalized Additive Models for Location Scale and Shape (GAMLSS) in R. *Journal of Statistical Software*. 2007;23:1 - 46.

## **Supplementary Tables and Figures**

### **Supplementary Table 1**

PRIORITIZE-HF study characteristics.

|  |  | **Placebo N=83** | **SZC N=86** |
| --- | --- | --- | --- |
| **Baseline** |  |  |  |
| Age, years | Mean ±SD | 70.9 ±8.0 | 72.7 ±8.8 |
| Sex | n (%) |  |  |
| Male |  | 53 (63.9) | 47 (54.7) |
| Race | n (%) |  |  |
| White |  | 82 (98.8) | 84 (97.7) |
| NYHA class | n (%) |  |  |
| II |  | 54 (65.1) | 55 (64.0) |
| III |  | 29 (34.9) | 31 (36.0) |
| BMI, kg/m2 | Mean ±SD | 30.3 ±5.7 | 29.6 ±5.8 |
| LVEF, % | Mean ±SD | 34.3 ±5.6 | 34.1 ±5.8 |
| KCCQ-OSS | Mean ±SD | 60.8 ±20.7 | 57.4 ±19.9 |
| Score = 0 | n (%) | 0 (0.0) | 0 (0.0) |
| Score = 100 | n (%) | 0 (0.0) | 1 (1.2) |
| KCCQ-TSS | Mean ±SD | 63.6 ±22.9 | 61.0 ±23.2 |
| Score = 0 | n (%) | 1 (1) | 0 (0) |
| Score = 100 | n (%) | 3 (3.6) | 7 (8.1) |
| KCCQ-CSS | Mean ±SD | 62.8 ±21.8 | 57.3 ±21.6 |
| Score = 0 | n (%) | 1 (1) | 0 (0) |
| Score = 100 | n (%) | 1 (1.2) | 1 (1.2) |
| **Follow-up** |  |  |  |
| Week 12 visit (Day 84), days from randomization | Min | 10 | 15 |
|  | Median | 80 | 83 |
|  | Max | 93 | 96 |
|  |  |  |  |
| KCCQ-OSS | Mean ±SD | 65.0 ±20.7 | 64.1 ±19.1 |
| Score = 0 | n (%) | 1 (1) | 0 (0.0) |
| Score = 100 | n (%) | 2 (2.6) | 0 (0.0) |
| KCCQ-TSS | Mean ±SD | 69.2±21.5 | 67.6 ±20.9 |
| Score = 0 | n (%) | 1 (1) | 0 (0) |
| Score = 100 | n (%) | 3 (3.8) | 5 (6.6) |
| KCCQ-CSS | Mean ±SD | 66.1 ±21.4 | 64.1 ±20.9 |
| Score = 0 | n (%) | 1 (1) | 0 (0) |
| Score = 100 | n (%) | 3 (3.8) | 1 (1.3) |
| BMI, Body Mass Index. | | | |


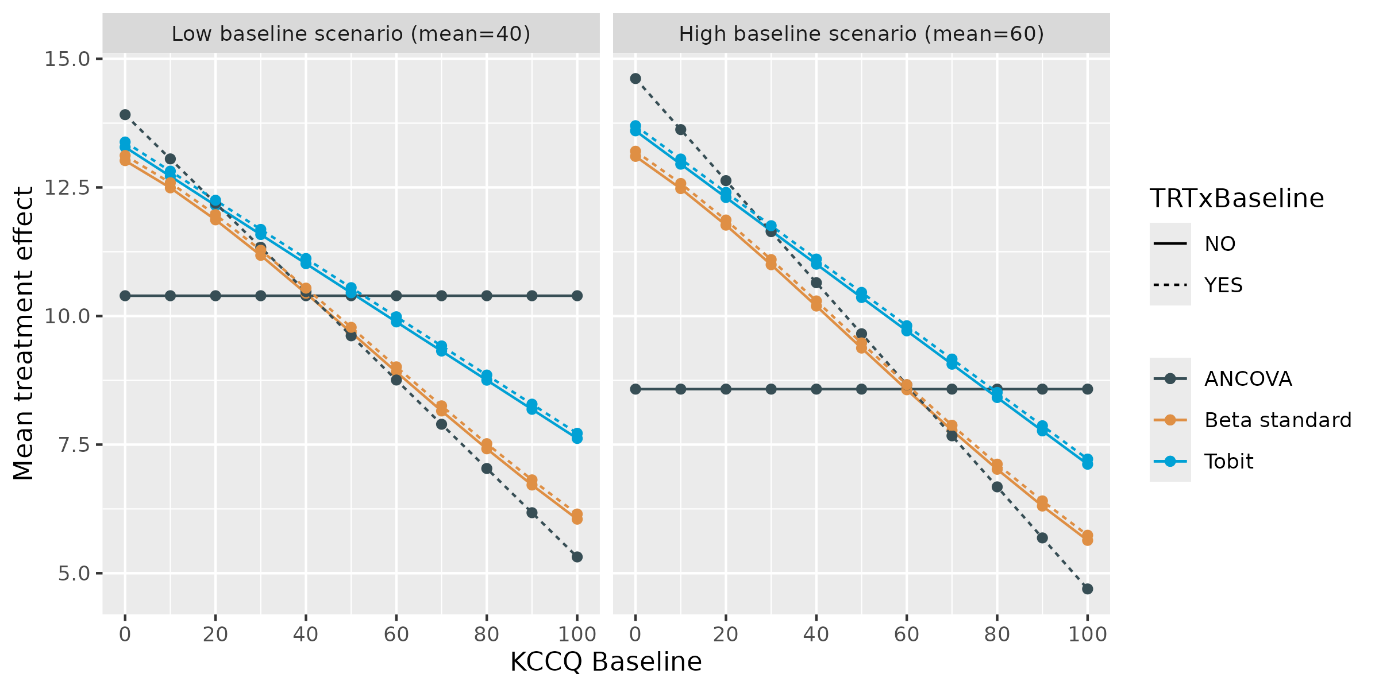


### **Supplementary Figure 1**

Mean treatment effects with/without treatment-by-baseline interaction terms for KCCQ change from baseline in simulated randomized trial. Results from two N=4000 simulated trials with a large effect on KCCQ and either a low or high baseline KCCQ distribution, respectively. Treatment effects represent differences in mean change from baseline between treatment groups. Mean effects > 0 favors SCZ treatment.


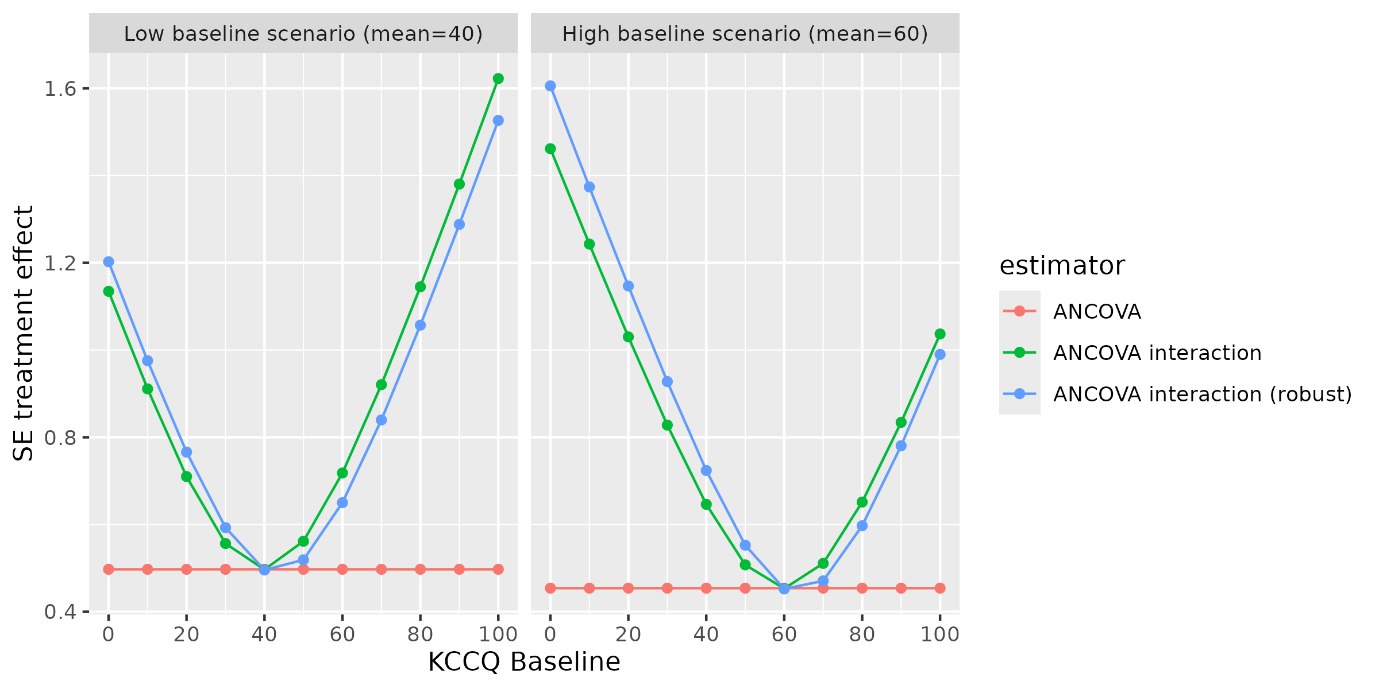


### **Supplementary Figure 2**

Standard errors of mean treatment effects in KCCQ change from baseline in simulated randomized trial, by ANCOVA models and SE estimators. Results from two N=4000 simulated trials with a large effect on KCCQ and either a low or high baseline KCCQ distribution, respectively. Treatment effects represent differences in mean change from baseline between treatment groups. Heteroskedasticity-consistent robust standard errors were estimated using the HC2 estimator.


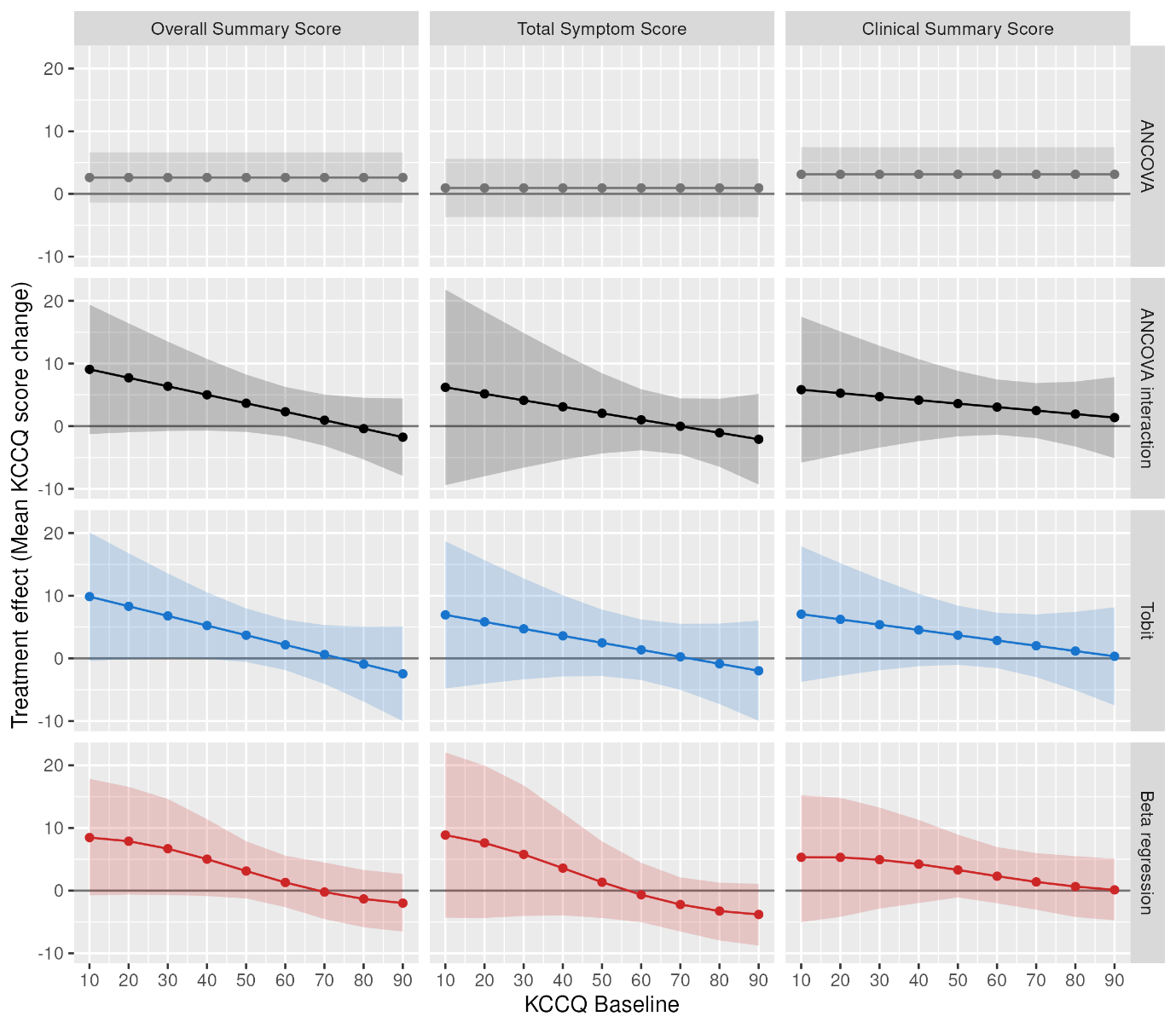


### **Supplementary Figure 3**

Mean treatment effects for KCCQ change from baseline in PRIORITIZE-HF for other KCCQ scores. Treatment effects represent differences in mean change from baseline at 12 weeks between treatment groups. Shaded areas represent 95% confidence intervals. Mean effects > 0 favors SCZ treatment.
